# Academic burnout in nursing students: An explanatory sequential design

**DOI:** 10.1002/nop2.1319

**Published:** 2022-08-18

**Authors:** Ali Asghar Ghods, Abbas Ebadi, Hamid Sharif Nia, Kelly‐Ann Allen, Tayyebeh Ali‐Abadi

**Affiliations:** ^1^ Nursing Care Research Center Semnan University of Medical Sciences Semnan Iran; ^2^ Behavioral Sciences Research Center, Lifestyle Institute, Nursing Faculty Baqiyatallah University of Medical Sciences Tehran Iran; ^3^ Department of Nursing, Amol Faculty of Nursing and Midwifery, Traditional and Complementary Medicine Research Center, Addiction Institute Mazandaran University of Medical Science Sari Iran; ^4^ School of Educational Psychology and Counseling, Faculty of Education Monash University Clayton Vic. Australia; ^5^ Centre for Wellbeing Science, The Melbourne Graduate School of Education The University of Melbourne Parkville Vic. Australia; ^6^ Department of Nursing Neyshabur University of Medical Sciences Neyshabur Iran

**Keywords:** burnout, nursing, student

## Abstract

**Aim:**

Despite the harmful effects of burnout among many nursing students, academic burnout is poorly understood. This study was conducted to better explain the concept of burnout in nursing students.

**Design:**

An explanatory sequential mixed‐method design was used.

**Methods:**

In the quantitative phase, the Maslach Burnout Inventory‐Student Survey was completed by 91 nursing students in eastern Iran. In the qualitative phase, individual interviews were conducted with 13 undergraduate nursing students, one nurse and one instructor. Data were analysed using the directed content analysis method.

**Results:**

Results from an ANOVA test showed differences in burnout scores in different semesters (*p* = .02) that were confirmed by the qualitative data. In addition to the three dimensions of the Maslach burnout model (exhaustion, cynicism and inadequacy feeling), qualitative data from the present study indicated the presence of a fourth dimension (incompatible learning style).

## 1 INTRODUCTION

The experience of burnout is usually accompanied by a range of emotional responses and experiences including emotional exhaustion, feelings of cynicism, and low professional efficacy (Maslach & Leiter, 2008; Schaufeli et al., 2002).

The experience of burnout influences workers from many different fields, but healthcare workers and nursing students, in particular, are exceedingly at risk of burnout (Galdino et al., 2020). Higher education systems are often accompanied by different levels of stress (Ali‐Abadi et al., 2020) that can often lead to academic burnout in students (Ríos‐Risquez et al., 2016). Because of this stress, medical students may feel exhausted or ineffective in their studies, which can lead to further feelings of burnout (Liu et al., 2018). Nursing education encountered critical challenges during the COVID‐19 pandemic, including changes to clinical placements and online classes—which have been found to influence burnout levels in students (Sveinsdóttir et al., 2021).

Evidence demonstrates that burnout leads to harmful consequences (Galdino et al., 2020), such as negative effects on the quality of nursing care, academic performance (da Silva et al., 2014) and withdrawal from courses (Tomaschewski‐Barlem et al., 2014). Therefore, further research on burnout in nursing students is vital (Sveinsdóttir et al., 2021).

### 1.1 Review of the literature

Burnout in nursing students is a problem experienced around the world. One Swedish study found the burnout rate among first‐year nursing students was 29.7% and for second year students it was 36.9% (Rudman & Gustavsson, 2012). Also, two meta‐analyses in developing countries found that over half of all medical students experienced academic burnout during their training courses (Hope & Henderson, 2014; IsHak et al., 2013). There is still a lack of research focusing on burnout among nursing students (Ríos‐Risquez et al., 2016), especially in Iran, despite evidence that the prevalence of burnout in Iranian medical student is markedly higher than other countries (Maghbouli et al., 2019). As such, analytical research conducted in Iran has shown that up to 49.2% of medical science students report higher academic burnout scores than the average general student population (Kharameh et al., 2016).

Several studies have investigated different dimensions of academic burnout in nursing students. Emotional exhaustion is often the first sign of academic burnout, and this can lead to a lack of effectiveness in academic performance (Charkhabi et al., 2013). Tomaschewski‐Barlem et al. (2013) identified emotional exhaustion, disbelief and low feelings ofself‐efficacy as dimensions of burnout among nursing students (Tomaschewski‐Barlem et al., 2013). Maslach and Leiter (2016) classified the dimensions of academic burnout into individual factors (emotional exhaustion), psycho‐social factors (cynicism, detatchment with peers and work) and low professional efficacy (feeling ineffective in typical responsibilities) (Maslach & Leiter, 2008). Research by Galdino et al. (2020) has also identfied emotional exhaustion, depersonalization, and reduced academic effectiveness as central dimensions of burnout (Galdino et al., 2020). According to previous studies, the variables that result in academic burnout may include personal intrinsic factors, interpersonal relationships and environmental factors [Deary, 2003 #2084] [Hwang, 2022 #2086] [Yang, 2016 #2087] but much of the research on academic burnout has been conducted with quantitative methods; however, qualitative research can provide a deeper understanding of the overall concept and greater detail of the aforementioned dimensions of burnout (Ochieng, 2009).

Overall, the literature reveals an incomplete understanding of nursing students' academic burnout among researchers (Schifferdecker & Reed, 2009). Mixed‐method approaches are needed to address this complex issue in a way that is more comprehensive than a purely qualitative or qualitative approach (Halcomb & Hickman, 2015). Therefore, the aim of this study was to identify dimensions of academic burnout in nursing students and its influential factors using a mixed‐methods design.

### 1.2 Research question

The following research questions were used to guide the present study:

#### 1.2.1 Qualitative question

1. What is the prevalence of academic burnout in nursing students?
2. What factors lead to burnout in nursing students?

#### 1.2.2 Qualitative question

1. What are the dimensions of burnout in nursing students?
2. Are similar dimensions of burnout found among nursing students with different academic and demographic backgrounds?

## 2 METHODS

### 2.1 Study design

The present study used an explanatory sequential mixed‐method approach that included an initial qualitative phase (content analysis) followed by a quantitative phase (cross‐sectional study). A mixed‐method approach was used to make use of the strengths of each form of data collection, while making up for the weaknesses of each (Speziale et al., 2011).

### 2.2 Target population and sampling procedure

In the quantitative phase, undergraduate nursing students with bachelor's degrees from Azad and public universities in Neyshabur completed the MBISS. The samples were selected using census sampling of 121 nursing students.

In the qualitative phase, purposeful sampling was used to collect qualitative data via interviews. The selection of students was based on the Maslach burnout model for in‐depth follow‐up, availability, willingness to participate, and the ability to communicate experiences in an articulate, expressive and reflective manner. Individual interviews were conducted with 15 nursing students who completed the questionnaire in the quantitative phase. Two of students dropped out of their courses and one was a conditional student.

### 2.3 Burnout measurement

In the quantitative phase, demographic information (i.e., age, gender, academic semester and marital status) and the level of academic burnout were evaluated by the Persian version of the MBISS after receiving permission from Schaufeli.

The Maslach Burnout Inventory‐Student Survey (MBISS) was selected for the present study as it is a modified form of the Maslach Burnout Inventory‐General Survey where irrelevant questions and words have been modified. Reliability and validity of this inventory in different samples have been reported as good (Gumz et al., 2013; Rostami et al., 2011). Also, it has been used in a number of studies in Iran to assess academic burnout (Elyasi et al., 2021; Sharififard et al., 2020). The 15‐item MBISS evaluates academic burnout across three dimensions: exhaustion (five items), cynicism (four items) and low professional efficacy (six items). All items are scored on a 7‐point Likert scale from 0 (never) to 6 (always). High scores in emotional exhaustion and cynicism and low efficacy scores represent greater academic burnout (Schaufeli et al., 2002). This scale has demonstrated acceptable reliability and validity from a number of studies conducted in Iran (Kharameh et al., 2016; Rostami et al., 2011). Coefficient Cronbach's alpha for exhaustion, cynicism and efficacy, respectively, were 88, 90 and 84 (Rostami et al., 2011).

### 2.4 Procedure

#### 2.4.1 Data collection

In the quantitative phase, data were collected by MBISS. In the qualitative phase, semi‐structured interviews were conducted individually in a quiet place at the university or educational class of the hospital. Each interview lasted between 40 and 60 minutes. The interviews aimed to identify the dimensions of academic burnout in nursing students and its influential factors. In order to increase variation sampling in the qualitative phase, students from different educational semesters and universities were selected. Due to some data from students' interviews being related to instructors and nurses, research team members interviewed one nursing instructor and one clinical nurse as well.

Based on the main categories from the Maslach burnout model and the results of the quantitative phase, the interview questions included probing questions and open‐ended questions about students' exhaustion, cynicism and academic performance, in addition to questions about factors causing academic burnout. The interviews were recorded, typed and coded by a nursing PhD candidate trained in qualitative research in order to limit response bias. Three experts in qualitative research conducted the interviews. The data collection continued until there was no new data from further interviews with other participants (Corbin & Strauss, 2014).

#### 2.4.2 Data analysis

In the quantitative phase, data were analysed by SPSS (version 18) using descriptive statistics. The higher‐than‐average score was selected for defining the amount of academic burnout (Schaufeli et al., 2002). Also, t‐test and analysis of variance (ANOVA) according to characteristics of the sample (academic and demographic) have been done to check for possible significant differences. Regarding low sample size, the G‐power software (version 3.0.10) was used to calculate the power of the results.

The unconstrained deductive content analysis method by Elo and Kyngäs (2008) was selected to analyse the qualitative data. To identify the latent and manifested data, researchers frequently reviewed and reflected on some questions: What is happening? Why are the participants giving these answers? The first researcher typed and uploaded the data in MAXQDA software (version 10). The interviews were read line by line inductively to extract initial codes. Next, a peer check was conducted by research team members. Finally, the extracted codes were allocated to the main and sub‐categories based on Maslach's matrix. New categories were extracted and grounded.

### 2.5 Trustworthiness

The trustworthiness of the data was evaluated by employing Ello's checklist (Elo et al., 2014). To decrease the threat of internal and external validities with the explanatory sequential mixed‐method design, the qualitative sample used the same individuals in the initial quantitative to explore the results in more depth (Creswell, 2014). Credibility was promoted by the researchers by validating the extracted codes and categories from interviews under the supervision of research team members who were experts in qualitative data collection. Trustworthiness of the data was promoted through the use of prolonged engagement, member checking, peer checking and maximum variation of sampling (Elo et al., 2014).

### 2.6 Ethics approval and consent to participate

The Semnan University of Medical Sciences' ethics committee approved this study (REDACTEDFse). Regarding the third COVID‐19 crisis and online education, surveys were sent via online survey software (Porsline). Participants who did not want to participate had the ability to opt‐out at any time. Prior to conducting the interviews, participants were verbally informed of the purpose of the research, data confidentiality and alternatives to participating. Participants' oral consent was given to opt‐in to the interview.

## 3 RESULTS

### 3.1 Quantitative phase

In this phase, 91 nursing students in different educational semesters participated. The average age of participants was 21.24 ± 1.34 years old (range: 18–25 years old) (Table 1).

**TABLE 1:** Descriptive characteristics of nursing students

Demographic variable	Number	Percent
Gender		
Female	42	46.2
Male	29	31.9
Missing data	20	22.1
Semester		
2	27	29.7
4	20	22
6	26	28.6
8	14	15.4
Missing data	4	4.3
Marital status		
Single	61	61.11
Married	10	11.11
Missing data	20	27.78

The quantitative phase results showed the mean scores from the different categories of the MBISS, including total burnout, 27.30 (*SD* = 10.38); emotional exhaustion, 7.21 (*SD* = 5.13); cynicism, 4.97 (*SD* = 4.31); and efficacy, 14.77 (*SD* = 6.34). The 50th percentile on the burnout was 28. Independent t‐tests indicated there was no significant difference in academic burnout among female and male students in the total burnout score and its sub‐scales. The same null result was found for marital status as well. The results of the ANOVA showed a significant statistical difference between the total score of burnout and educational semesters (*p* = .02) (Table 2). This was represented by a good effect, *d* = 0.4. Least Significant Difference test (LSD) indicated there was a significant difference in burnout scores between semesters 2 and 4.

**TABLE 2:** Mean burnout and its sub‐scales among nursing students according to semester

Variable	Exhaustion	Cynicism	Low professional efficacy	Total burnout
Semester				
2	5.22 ± 4.45	3.77 ± 4.30	13.30 ± 5.44	22.73 ± 9.90
4	9.25 ± 5.05	6.85 ± 3.93	15.94 ± 6.81	32.15 ± 9.17
6	7.96 ± 4.72	4.65 ± 4.20	14.91 ± 6.09	27.69 ± 10.25
8	6.30 ± 6.70	5 ± 5.20	16 ± 8.50	28.00 ± 12.24
*p*‐value	.04	.12	.5	.02

### 3.2 Qualitative phase

The qualitative phase was conducted by researchers who had an in‐depth understanding of the different dimensions of academic burnout and what factors influence burnout. In this phase, eight sub‐categories and four main categories were identified as academic burnout dimensions among nursing students. The main categories included exhaustion, cynicism, low professional efficacy and incompatible learning style (Table 3).

**TABLE 3:** Unit meanings, sub‐categories and categories extracted from qualitative data

Unit meaning	Subcategory	Category
*We had Anatomy course in our first semester also Physiology. I had Fundamentals of Nursing in 4 units and a Biochemistry course. All these courses were so hard. The educational managers could select more simple courses for our first semester for being familiar with the nursing setting*. (Female, semester 5) *We had biology before entering the university with more simple abbreviations, once in physiology we had confronted difficult abbreviations. In the Anatomy and Physiology course, we were always shocked*. (Male, semester 3)	Academic assignments	Exhaustion
*The students have classes in mornings. They have placement in the evenings. They are so tired due to their morning activities. The students tell us that other placements start without any rest*”. (Female, Instructor) *Hospital employers consider students as labor personnel instead of teaching them*. (Male, semester 5)	Clinical work pressure	
*At first, I start designing a study plan for my lessons during the term, but I didn't complete it to final process at the end of term …*. *The professor started teaching while we sat on round tables, and we do not listen and sometimes took a nap*. (Male, semester 7)	Theoretical lessons	Cynicism
*I was telling some information to the patient, but he avoids listening and rejects that. Sometimes these events make us less energetic that the patient doesn't pay attention to us. I say that I did my duty to explain to him, but he doesn't want to listen*. (Female, semester 8) *Hospital staff do more important activities like nursing reports by themselves, simple unimportant tasks left for students. I don't like to do those tasks*. 5^th^ semester male student	Clinical activities	
*In the first semester, we were always scared of everything …Can I be successful in this major*. (Female, semester 3). *Can I really do this task or the circumstance was not appropriate to do? Is it always in this form, and I am unable to do this*? (Female, semester 8)	Inadequacy in patient caring	Low professional efficacy
*I was stressed when I didn't study enough. I think in exams we made stress ourselves. We don't study the books during the term while we left them for exam night at once. On the exam night, you read the book once and you are stressed for incomplete understanding*. (Male, semester 7) *Studying in universities is completely different from the high school studying process. This increases our educational failure*. (Female, semester 3)	Lack of educational progress	
*I am not interested in practical tasks while I am skillful in mental and research‐based activities*. (Dropout student).	Interest in practical tasks	Incompatible learning style
*Some information is helpful for us in the hospital because they are useable. I think a usable course is the helpful one for our hospital tasks. We are working in hospitals that should be the knowledge usage*. (Conditional student, semester 3)	Applicability of knowledge	

#### 3.2.1 Exhaustion

Exhaustion, in part due to academic pressure, was one of the main dimensions noted by participants. This dimension included two sub‐categories: academic assignments and clinical work pressure.

##### 3.2.1.1 Academic assignments

Almost all junior students made note of feelings of exhaustion caused by the start of the academic year. They stated that the first course units were not usually simple to get familiar with, and they could not adapt to the nursing environment. Some students discussed the pressure of memorizing difficult abbreviations and acronyms in their first semester and were shocked by some difficult abbreviations.

##### 3.2.1.2 Clinical work pressure

The pressure of clinical training courses was one of the main factors influencing nursing students' exhaustion. Some students, and one instructor, talked about the pressure of their training programmes. They were exhausted from condensed internship plans; in addition, they were considered as staff during hospital internships.

#### 3.2.2 Cynicism

Students indicated their cynicism by pointing to a lack of interest in lessons and clinical activities and being absent in educational plans. This main category included two sub‐categories: theoretical lessons and clinical activities.

##### 3.2.2.1 Theoretical lessons

Students noted being less motivated about theoretical lessons. A number of students spoke about their lack of attention and lack of understanding of the theoretical content.

##### 3.2.2.2 Clinical activities

Some students declared that they were unmotivated for clinical placements because the patients ignored the importance of their time and wasted their energy.

#### 3.2.3 Low professional efficacy

The third dimension of academic burnout in nursing students was their feeling of incompetence in completing their nursing tasks. This inefficacy category was broken into two sub‐categories: inadequacy in patient care and a lack of educational progress.

##### 3.2.3.1 Inadequacy in patient care

Inefficacy in caring for patients was declared by almost all junior students. They experienced feelings of failure regarding patient care and fears of the unknown consequences resulting from feelings of inefficacy.

##### 3.2.3.2 Lack of educational progress

The students noted that a lack of planning and commitment to their education progress mainly resulted in low grades and therefore feelings of inefficacy.

#### 3.2.4 Incompatible learning style

The fourth category that emerged was incompatible learning style made up of two sub‐categories: interest in practical tasks and the applicability of knowledge. Almost all students thought about the impact their studies would have on their job expectations and wanted practical applications for their knowledge.

## 4 DISCUSSION

The aim of the present study was to explain and explore the dimensions and influential factors of academic burnout in nursing students.

The results of the quantitative phase of the study found burnout values greater than the 50th percentile indicating that nursing students had higher levels of burnout than the national average, whereas those less than the 50th percentile were better off. This result is in acconcordance with international research that has indicated burnout scores in nursing students (and the associated dimensions) were higher than the average scores in the general population (dos Santos Boni et al., 2018; Galdino et al., 2020; IsHak et al., 2013; Santen et al., 2010). The amount of academic burnout found in other studies has ranged from 21% to 71% (IsHak et al., 2013; Santen et al., 2010) which may be related to the level of support, stress and control over one's life (Santen et al., 2010; Wilks, 2008).

One of the main results of the quantitative phase was the evidence of academic burnout among junior nursing students over senior nursing students. Nursing students in Semesters 2 and 4 experienced more academic burnout in comparison with other semesters. These results are similar to Ferri et al.'s (2015) findings, where younger nursing students had higher levels of burnout due to the transition period between their idealistic expectations and daily practice (Ferri et al., 2015). However, the sample size in the present study was small which may have impacted the result. As is evident, inappropriate experiences will increase occupational pressures even after completion of a course of study. Wilks (2008) also reported more than 27% of nursing students withdrawing in their first academic year (Wilks, 2008). The qualitative data also indicated that the 2‐ and 4‐semester students encountered a higher degree of academic burnout in the exhaustion dimension. It can be related to the workload of the courses in comparison with other students. Nursing students need to pass 130 educational units in theoretical classes and training programmes (Education, 2020). The high ranges of theoretical studies are related to the first semester of education, and students in 7 and 8 semesters only need to pass 18 training courses. Dehghan‐nayeri & Adib‐Hajbaghery, 2011 also indicate this heavy load of activities for nursing students in universities (Dehghan‐nayeri & Adib‐Hajbaghery, 2011). Another reason for this issue may be the start of clinical courses. It is essential for nursing students to get more experience from clinical settings and workplaces for senior students (Aqila et al., 2019). For the majority of students, continuing academic education would be considered as a transition period that needs further adaptation to educational settings (Wilks, 2008). Nursing students enter clinical settings from their third semester; experiencing more challenges in this context would increase their academic burnout (Ching et al., 2020).

The qualitative phase mostly aligned with Maslach's burnout model and explored the various dimensions of academic burnout, including exhaustion, cynicism, low professional efficacy and incompatible learning style (Maslach & Leiter, 2008). The concept of incompatible learning style was an additional dimension found in the present study that was not a component of Maslach's original model.

One of the main dimensions of burnout in this research was exhaustion. Activity load was an influential environmental factor for explaining academic burnout. When people feel their valuable sources are used for useless activities without receiving any positive feedback, their mental and physical condition will gradually change (Yang, 2004). In this regard, students feel overwhelmed when they encounter various problems due to time constraints during their learning process. These results aligned with Alzayyat & Al‐Gamal, 2014, Yang (2004), and Dugué et al. (2018). In contrast to the results of this study, Tomaschewski‐Barlem et al. (2014) found that despite experiencing high levels of exhaustion, students did not experience burnout (Tomaschewski‐Barlem et al., 2014). However, this may be due to the close relationship students reported having with their instructors in that study and their high motivation to become a nurse (Tomaschewski‐Barlem et al., 2014).

Another dimension of academic burnout found in the qualitative phase was cynicism, classified more specifically as a lack of interest in participating in theoretical classes and clinical training. In accordance with Galdino's study, cynicism is a psychological adaptation that students use to deal with suffering from their heavy academic load and workload (Galdino et al., 2020). Academic engagement is a significant variable that leads to improvements in educational achievement, educational satisfaction and well‐being in students (Dugué & Dosseville, 2018). The results of the present study also indicated that personal determination and enjoyable internship experiences would be influential for continuing education in students. Academic burnout can lead to more absences, less motivation for educational activities and a greater likelihood of withdrawal from nursing education (Yang, 2004). One of the reasons for cynicism and a lack of interest in their major would be the students' lack of understanding regarding academic expectations and instructors' demands (dos Santos Boni et al., 2018). Students noted that courses focused more on theory than practical knowledge were frustrating, as the educational content was not related to the identity of the major and not usable. This resulted in low professional efficacy and feelings that they were wasting time and energy (Aghajari et al., 2018). Borhani et al. also indicated that having less informative knowledge about the goals and difficult circumstances of the nursing profession would result in less motivated students (Borhani et al., 2010). Another reason for this lack of interest may be due to weak relationships between students and professors or hospital employers (Babenko‐Mould & Laschinger, 2014; Pryjmachuk & Richards, 2007). Effective relationships with university professors through consultations, receiving feedback and encouragement are important for providing a positive perspective for students to be become successful nurses (Aghajari et al., 2018).

A feeling of inefficacy was another dimension of burnout in this study. It was noted as a lack of educational progress and incompetence in caring for patients. Nurses' lack of educational success has been found across the world in both developed and developing nations (Lancia et al., 2013; Liu et al., 2015; Pryjmachuk & Richards, 2007). In the same way, Mikaeili et al.'s study also indicated that there is a relationship between academic burnout and decreased educational success. The students who experience academic burnout feel disappointed, sensitive, depressed and less interested in their academic lessons (Mikaeeli et al., 2013). Similar to other studies, a lack of efficacy in patient care was demonstrated by students' sense of responsibility for what might happen to patients mixed with their fear of making mistakes (Liu et al., 2015; Moridi et al., 2014). Alzayyat et al.'s review of the literature also signified the fear of committing mistakes in clinical settings among nursing students (Alzayyat & Al‐Gamal, 2014).

Finally, incompatible learning style was another dimension of academic burnout which was found in the present study but was not noted in Maslach's model. Learning style is a preferred behaviour for acquiring knowledge, skills, and attitudes through study or experience (Kolb, 2007). In a national research study by Aalaa et al., an accommodative learning style was found to be the dominating learning style of nursing students. The accommodating learning style is “hands‐on” (Aalaa et al., 2014). As learning style is one of the important factors for learning, incompatibility with learning style in nursing students would increase their disappointment, increase educational failure and make them less motivated—sometimes resulting in academic dropout (Azizi et al., 2002; Li et al., 2008; Rassool & Rawaf, 2007). Also, Samra et al showed a mismatch in learning style may be a cause of academic burnout (McManus et al., 2004).

### 4.1 Limitations

A main theoretical limitation of this study is the use of a simple sampling process during the quantitative phase, which decreases the data's generalizability. Although students' GPA was another factor predicting burnout, it was not asked during the quantitative phase. In addition, as some variables, including age, were not controlled, results should be interpreted cautiously. Also, as burnout was only explored in nursing students, assessing all medical science students is recommended. Future quantitative studies should focus on determining burnout‐reducing interventions using the extracted factors found in this study as a guideline.

## 5 CONCLUSION

In this explanatory mixed‐method study, Maslach's theory guided the quantitative and qualitative data collection to assess and explore the concept of burnout in nursing students. The reported burnout in nursing students in the study was higher than the mean in this population, and burnout scores between semesters 2 and 4 showed a significant difference. Also, the current study showed incompatible learning style as an additional fourth dimension of burnout. Finally, the differences in burnout score in various semesters was explained in depth in the qualitative phase.

Evidence from the present study suggests that educational training managers should implement interventions to promote resilience (da Silva et al., 2014), social support, relaxation skills and confidence in future competence to reduce academic burnout (Youssef, 2016), students' mental disorders and academic withdraws (Schifferdecker & Reed, 2009). Moreover, recognizing learning strategies would be a helpful step in understanding and developing the concept of academic burnout based on those studies that showed a relationship between learning style and academic burnout (Çakır et al., 2014; McManus et al., 2004). Regarding this issue, authorities could consider learning perferences when engaging the student during the training process as well as redesigning the curriculum based on the dominant learning style in that setting to prepare them for providing better quality care.

## CONFLICT OF INTEREST

None.

## Data Availability

Data are available through the corresponding author.
